# 1-(6-Ferrocenylhex­yl)-1*H*-imidazole

**DOI:** 10.1107/S1600536810007737

**Published:** 2010-03-17

**Authors:** Vincent O. Nyamori, Muhammad D. Bala, Demetrius C. Levendis

**Affiliations:** a School of Chemistry, University of KwaZulu-Natal, Westville Campus, Private Bag X54001, Durban 4000, South Africa; b Structural Chemistry Unit, School of Chemistry, University of the Witwatersrand, Johannesburg 2050, South Africa

## Abstract

The title compound, [Fe(C_5_H_5_)(C_14_H_19_N_2_)], is characterized by a ferrocenyl group separated from an imidazole functionality by a straight-chain hexyl unit. The two cyclo­penta­dienyl rings of the ferrocenyl group show a marginal inward tilt of 2.17 (2)°. The imidazole unit, which is essentially planar (with a maximum deviation of 0.007 A for one of the N atoms) and tilted away from the ferrocenyl group [dihedral angle between the substituted ferrocenyl ring and the imidazole = 122.6 (1)°], is involved in inter­molecular C—H⋯N inter­actions.

## Related literature

For related structures, see: Hua *et al.* (2004 ▶); Nyamori & Bala (2008 ▶).
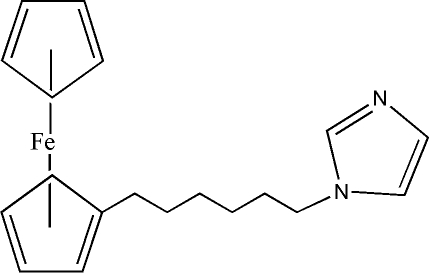


## Experimental

### 

#### Crystal data

[Fe(C_5_H_5_)(C_14_H_19_N_2_)]
*M* *_r_* = 336.25
Orthorhombic, 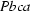

*a* = 15.587 (3) Å
*b* = 7.6042 (12) Å
*c* = 27.773 (4) Å
*V* = 3291.9 (9) Å^3^
*Z* = 8
Mo *K*α radiation
μ = 0.91 mm^−1^
*T* = 173 K
0.40 × 0.24 × 0.02 mm

#### Data collection

Bruker SMART APEXII CCD area-detector diffractometer
Absorption correction: multi-scan (*SADABS*; Bruker, 2005 ▶) *T*_min_ = 0.666, *T*_max_ = 0.751
24220 measured reflections
3061 independent reflections
2017 reflections with *I* > 2σ(*I*)
*R*_int_ = 0.121

#### Refinement

*R*[*F* ^2^ > 2σ(*F* ^2^)] = 0.042
*wR*(*F* ^2^) = 0.101
*S* = 0.98
3061 reflections
199 parameters
H-atom parameters constrained
Δρ_max_ = 0.27 e Å^−3^
Δρ_min_ = −0.44 e Å^−3^

### 

Data collection: *APEX2* (Bruker, 2005 ▶); cell refinement: *SAINT-NT* (Bruker, 2005 ▶); data reduction: *SAINT-NT* (Bruker, 2005 ▶); program(s) used to solve structure: *SHELXTL* (Sheldrick, 2008 ▶); program(s) used to refine structure: *SHELXTL*; molecular graphics: *ORTEP-3* (Farrugia, 1997 ▶); software used to prepare material for publication: *SHELXTL*.

## Supplementary Material

Crystal structure: contains datablocks global, I. DOI: 10.1107/S1600536810007737/dn2540sup1.cif

Structure factors: contains datablocks I. DOI: 10.1107/S1600536810007737/dn2540Isup2.hkl

Additional supplementary materials:  crystallographic information; 3D view; checkCIF report

## Figures and Tables

**Table 1:** Hydrogen-bond geometry (Å, °)

*D*—H⋯*A*	*D*—H	H⋯*A*	*D*⋯*A*	*D*—H⋯*A*
C19—H19⋯N2^i^	0.93	2.58	3.399 (5)	147

Symmetry code: (i) 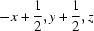.

**Fig. 1.**
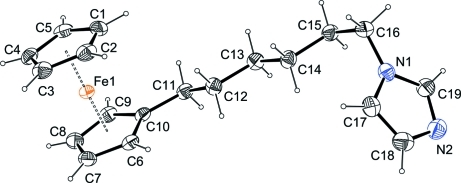
Molecular structure of the title compound with the atom numbering scheme. Displacement ellipsoids are drawn at the 30% probability level. H atoms are presented as small spheres of arbitrary radius.

## supplementary crystallographic information

### Comment

The title compound (I) was prepared as part of our study into the development of imidazole based ionic liquids and precursors to *N*-heterocyclic carbene ligands bearing a ferrocenyl functionality. Related compounds bearing structural similarities to (I) have been communicated by Hua *et al.* (2004) and Nyamori & Bala (2008). The compound (I) shows a fairly linear alkyl carbon-carbon bond linkage of the ferrocenyl moiety with the imidazole. This is evident from the torsion angles; C10—C11—C12—C13 = -178.3 (3)°, C12—C13—C14—C15 = -177.4 (3)° and C13—C14—C15—C16 = 178.3 (3)°. The average bond distances between the carbon atoms (C11 to C15) is 1.5375 (Å) which is normal for saturated alkyl carbon-carbon bonds. The bond distance between C16—N1 is 1.450 (4) Å as expected. The imidazole ring slightly deviates from planarity as indicated by the torsion angles; N1—C19—N2—C18 = -1.3 (4)° and C17—C18—N2—C19 = 1.2 (4)°. The bond length between C19—N2 is 1.312 (4) Å which is a clear indication of a localized carbon-carbon double bond while those of C18—N2 = 1.379 (4) Å, C17—C18 = 1.361 (5) Å and C17—N1 = 1.365 (4) Å are longer and indicate delocalization of π electrons. This implies that aromaticity of the imidazole ring is also reduced. The torsion angles within the atoms of the ferrocenyl rings indicate that the rings are fairly planar. The two ferrocenyl rings also show a marginal tilt towards each other with an angle of 2.17°. The average bond distance of substituted ferrocenyl ring carbon atoms and the metal centre (Fe1) is found to be 2.039 (3) Å while that of the unsubstituted ferrocenyl ring is obtained as 2.027 (3) Å. A value of 114.0 (3)° is observed for the N1—C16—C15 angle. The dihedral angle between the substituted ferrocenyl ring and the imidazole is found to be 122.6 (1)°. These observations confirm that the molecule is kinked and this, in turn, affects the molecular packing of the molecules which are held together by weak C—H···N interactions with a contact distance of about 2.583 Å.

### Experimental

Imidazole(148 mg, 2.17 mmol) was added to acetone (2.0 cm^3^) and the solution was stirred. Potassium hydroxide powder (128 mg, 2.27 mmol) was then introducedand allowed to dissolve, forming a homogenous solution. The solution was stirred for about 30 minutes and then 6-bromohexylferrocene (834 mg, 2.39 mmol) in acetone (1.0 cm^3^) was introduced to the solution dropwise and allowed to stir for about 1 hour at room temperature. The solution was then filtered and concentrated. The crude product was passed through a column of silica gel. Diethyl ether recovered unreacted 6-bromohexylferrocene (150 mg)while ethyl acetate/methanol (10:1) afforded the product as yellow crystals (591 mg, 81 %); mp 68-70°C;IR (KBr cm^-1^) 3090, 2932, 2859, 1655, 1508, 1466, 1439, 1234, 1107,1076, 999, 829, 802, 745, 671, 509, 486; ^1^H NMR (CDCl_3_)7.47 (1*H*, s, NCH), 7.07 (1*H*, s, NCH), 6.91 (1*H*, s, NCH), 4.09 (5*H*, s, C_5_H_5_),4.04 (4*H*, m, C_5_H_4_), 3.91 (2*H*, t, J 7.1, CH_2_), 2.31 (2*H*, t, J 7.3, CH_2_), 1.77 (2*H*, m, CH_2_), 1.48 (2*H*,m, CH_2_), 1.31 (4*H*, m, 2 x CH_2_); ^13^C NMR (CDCl_3_)137.48, 129.76, 119.22, 89.45, 68.87, 68.45, 67.48, 47.42, 31.44, 31.37, 29.89,29.35, 26.83; m/z (EI) 337 (M^+^+1, 14%), 336 (M^+^, 61%),272 (19), 271 (100), 269 (10), 199 (9), 121 (26); Anal. Calc for C_19_H_24_N_2_Fe:*C*, 67.8; H, 7.2; N, 8.3; [M^+^], 336.128888. Found: C, 67.5; H, 7.3;N, 8.0; [M^+^], 336.128905.

### Refinement

All H-atoms were refined using a riding model, with C—H = 0.93 Å and *U_i_*_so_(H) = 1.2*U*_eq_(C) for aromatic, C—H = 0.97 Å and *U_i_*_so_(H) = 1.2*U*_eq_(C) for CH_2_, C—H = 0.96 Å and *U_i_*_so_(H) = 1.5*U*_eq_(C) for CH_3_, N—H = 0.86 Å and *U_i_*_so_(H) = 1.2*U*_eq_(N) for NH, and O—H = 0.82 Å and *U_i_*_so_(H) = 1.5*U*_eq_(O) for OH.

### Crystal data

**Table:**

[Fe(C_5_H_5_)(C_14_H_19_N_2_)]	*F*(000) = 1424
*M**_r_* = 336.25	*D*_x_ = 1.357 Mg m^−^^3^
Orthorhombic, *P**b**c**a*	Mo *K*α radiation, λ = 0.71073 Å
Hall symbol: -P 2ac 2ab	θ = 1.5–25.5°
*a* = 15.587 (3) Å	µ = 0.91 mm^−^^1^
*b* = 7.6042 (12) Å	*T* = 173 K
*c* = 27.773 (4) Å	Plate, yellow
*V* = 3291.9 (9) Å^3^	0.40 × 0.24 × 0.02 mm
*Z* = 8	

### Data collection

**Table:**

Bruker SMART CCD area-detector diffractometer	3061 independent reflections
Radiation source: fine-focus sealed tube	2017 reflections with *I* > 2σ(*I*)
graphite	*R*_int_ = 0.121
φ and ω scans	θ_max_ = 25.5°, θ_min_ = 1.5°
Absorption correction: multi-scan (*SADABS*; Bruker, 2005)	*h* = −18→18
*T*_min_ = 0.666, *T*_max_ = 0.751	*k* = −9→8
24220 measured reflections	*l* = −33→33

### Refinement

**Table:**

Refinement on *F*^2^	Primary atom site location: structure-invariant direct methods
Least-squares matrix: full	Secondary atom site location: difference Fourier map
*R*[*F*^2^ > 2σ(*F*^2^)] = 0.042	Hydrogen site location: inferred from neighbouring sites
*wR*(*F*^2^) = 0.101	H-atom parameters constrained
*S* = 0.98	*w* = 1/[σ^2^(*F*_o_^2^) + (0.0515*P*)^2^] where *P* = (*F*_o_^2^ + 2*F*_c_^2^)/3
3061 reflections	(Δ/σ)_max_ < 0.001
199 parameters	Δρ_max_ = 0.27 e Å^−^^3^
0 restraints	Δρ_min_ = −0.44 e Å^−^^3^

### Special details

**Table:**

Geometry. All esds (except the esd in the dihedral angle between two l.s. planes) are estimated using the full covariance matrix. The cell esds are taken into account individually in the estimation of esds in distances, angles and torsion angles; correlations between esds in cell parameters are only used when they are defined by crystal symmetry. An approximate (isotropic) treatment of cell esds is used for estimating esds involving l.s. planes.
Refinement. Refinement of *F*^2^ against ALL reflections. The weighted *R*-factor wR and goodness of fit *S* are based on *F*^2^, conventional *R*-factors *R* are based on *F*, with *F* set to zero for negative *F*^2^. The threshold expression of *F*^2^ > σ(*F*^2^) is used only for calculating *R*-factors(gt) etc. and is not relevant to the choice of reflections for refinement. *R*-factors based on *F*^2^ are statistically about twice as large as those based on *F*, and *R*- factors based on ALL data will be even larger.

### Fractional atomic coordinates and isotropic or equivalent isotropic displacement parameters (Å^2^)

**Table:**

	*x*	*y*	*z*	*U*_iso_*/*U*_eq_	
C1	0.0402 (2)	0.4449 (4)	0.43353 (12)	0.0507 (9)	
H1	0.0651	0.4976	0.4603	0.061*	
C2	−0.0306 (2)	0.3298 (4)	0.43400 (12)	0.0508 (9)	
H2	−0.0611	0.2938	0.4611	0.061*	
C3	−0.0470 (2)	0.2788 (4)	0.38591 (13)	0.0508 (9)	
H3	−0.0902	0.2031	0.3757	0.061*	
C4	0.0136 (2)	0.3629 (4)	0.35620 (12)	0.0463 (8)	
H4	0.0175	0.3516	0.3229	0.056*	
C5	0.0669 (2)	0.4665 (4)	0.38522 (13)	0.0493 (8)	
H5	0.1119	0.5369	0.3746	0.059*	
C6	0.10463 (19)	−0.0075 (4)	0.44559 (12)	0.0410 (8)	
H6	0.0737	−0.0490	0.4719	0.049*	
C7	0.0912 (2)	−0.0557 (4)	0.39673 (12)	0.0493 (9)	
H7	0.0501	−0.1343	0.3854	0.059*	
C8	0.1513 (2)	0.0373 (4)	0.36845 (12)	0.0499 (9)	
H8	0.1568	0.0307	0.3352	0.060*	
C9	0.20114 (19)	0.1412 (5)	0.39933 (11)	0.0448 (8)	
H9	0.2453	0.2155	0.3897	0.054*	
C10	0.17368 (18)	0.1155 (4)	0.44742 (11)	0.0368 (7)	
C11	0.21197 (19)	0.1987 (4)	0.49179 (11)	0.0439 (8)	
H11A	0.2667	0.1426	0.4983	0.053*	
H11B	0.2233	0.3216	0.4849	0.053*	
C12	0.15795 (19)	0.1885 (4)	0.53670 (10)	0.0438 (8)	
H12A	0.1481	0.0658	0.5445	0.053*	
H12B	0.1026	0.2419	0.5302	0.053*	
C13	0.1980 (2)	0.2788 (4)	0.58019 (11)	0.0464 (8)	
H13A	0.2554	0.2331	0.5849	0.056*	
H13B	0.2028	0.4037	0.5736	0.056*	
C14	0.14718 (19)	0.2535 (4)	0.62592 (11)	0.0445 (8)	
H14A	0.1445	0.1287	0.6330	0.053*	
H14B	0.0890	0.2938	0.6204	0.053*	
C15	0.1825 (2)	0.3479 (4)	0.66974 (10)	0.0450 (8)	
H15A	0.1868	0.4724	0.6625	0.054*	
H15B	0.2400	0.3048	0.6761	0.054*	
C16	0.1286 (2)	0.3249 (4)	0.71481 (12)	0.0491 (9)	
H16A	0.1496	0.4046	0.7394	0.059*	
H16B	0.0699	0.3578	0.7076	0.059*	
C17	0.0772 (2)	0.0108 (5)	0.72108 (11)	0.0513 (9)	
H17	0.0329	0.0137	0.6987	0.062*	
C18	0.1031 (2)	−0.1306 (5)	0.74738 (13)	0.0577 (9)	
H18	0.0784	−0.2419	0.7461	0.069*	
C19	0.1837 (2)	0.0833 (5)	0.76738 (11)	0.0496 (8)	
H19	0.2255	0.1507	0.7825	0.060*	
N1	0.12910 (15)	0.1475 (4)	0.73396 (9)	0.0434 (7)	
N2	0.17139 (18)	−0.0843 (4)	0.77628 (10)	0.0572 (7)	
Fe1	0.07515 (2)	0.20943 (5)	0.405519 (14)	0.03346 (15)	

### Atomic displacement parameters (Å^2^)

**Table:**

	*U*^11^	*U*^22^	*U*^33^	*U*^12^	*U*^13^	*U*^23^
C1	0.055 (2)	0.041 (2)	0.057 (2)	0.0156 (17)	−0.0107 (18)	−0.0145 (17)
C2	0.051 (2)	0.048 (2)	0.054 (2)	0.0168 (17)	0.0168 (17)	0.0046 (17)
C3	0.0330 (16)	0.046 (2)	0.074 (2)	0.0070 (15)	−0.0059 (16)	−0.0014 (19)
C4	0.0495 (19)	0.046 (2)	0.0437 (19)	0.0134 (17)	−0.0033 (16)	0.0051 (16)
C5	0.048 (2)	0.0306 (18)	0.069 (2)	0.0056 (15)	−0.0020 (18)	0.0077 (17)
C6	0.0401 (17)	0.0340 (18)	0.049 (2)	0.0011 (14)	0.0006 (14)	0.0104 (16)
C7	0.048 (2)	0.0349 (19)	0.065 (2)	0.0036 (15)	−0.0140 (17)	−0.0059 (17)
C8	0.053 (2)	0.049 (2)	0.048 (2)	0.0177 (17)	0.0018 (17)	−0.0085 (17)
C9	0.0358 (16)	0.0439 (19)	0.055 (2)	0.0078 (14)	0.0064 (15)	0.0071 (17)
C10	0.0357 (16)	0.0313 (17)	0.0433 (19)	0.0038 (14)	−0.0035 (14)	0.0061 (15)
C11	0.0367 (16)	0.0397 (19)	0.055 (2)	−0.0007 (14)	−0.0069 (14)	0.0066 (16)
C12	0.0376 (17)	0.049 (2)	0.0451 (19)	−0.0048 (15)	−0.0033 (14)	0.0016 (16)
C13	0.0424 (18)	0.046 (2)	0.0505 (19)	−0.0038 (15)	−0.0007 (15)	0.0042 (16)
C14	0.0409 (17)	0.045 (2)	0.048 (2)	−0.0015 (15)	−0.0062 (15)	0.0029 (15)
C15	0.0459 (18)	0.0388 (19)	0.050 (2)	−0.0022 (15)	−0.0051 (15)	0.0022 (16)
C16	0.054 (2)	0.041 (2)	0.053 (2)	0.0070 (15)	−0.0014 (17)	−0.0021 (16)
C17	0.0445 (19)	0.063 (2)	0.0463 (19)	−0.0051 (18)	0.0012 (17)	−0.0018 (18)
C18	0.061 (2)	0.053 (2)	0.059 (2)	−0.0041 (19)	0.0139 (19)	−0.003 (2)
C19	0.0413 (18)	0.056 (2)	0.052 (2)	0.0039 (17)	−0.0037 (16)	−0.0029 (18)
N1	0.0406 (15)	0.0482 (17)	0.0413 (16)	0.0016 (13)	0.0005 (12)	−0.0042 (13)
N2	0.0596 (19)	0.058 (2)	0.0542 (18)	0.0079 (16)	0.0053 (15)	0.0055 (16)
Fe1	0.0323 (2)	0.0293 (2)	0.0388 (3)	0.00365 (19)	0.00101 (19)	0.0008 (2)

### Geometric parameters (Å, °)

**Table:**

C1—C2	1.409 (5)	C10—C11	1.508 (4)
C1—C5	1.414 (4)	C10—Fe1	2.055 (3)
C1—Fe1	2.027 (3)	C11—C12	1.507 (4)
C1—H1	0.9300	C11—H11A	0.9700
C2—C3	1.414 (5)	C11—H11B	0.9700
C2—Fe1	2.044 (3)	C12—C13	1.523 (4)
C2—H2	0.9300	C12—H12A	0.9700
C3—C4	1.408 (4)	C12—H12B	0.9700
C3—Fe1	2.049 (3)	C13—C14	1.509 (4)
C3—H3	0.9300	C13—H13A	0.9700
C4—C5	1.401 (4)	C13—H13B	0.9700
C4—Fe1	2.039 (3)	C14—C15	1.517 (4)
C4—H4	0.9300	C14—H14A	0.9700
C5—Fe1	2.039 (3)	C14—H14B	0.9700
C5—H5	0.9300	C15—C16	1.518 (4)
C6—C7	1.421 (4)	C15—H15A	0.9700
C6—C10	1.427 (4)	C15—H15B	0.9700
C6—Fe1	2.042 (3)	C16—N1	1.450 (4)
C6—H6	0.9300	C16—H16A	0.9700
C7—C8	1.412 (5)	C16—H16B	0.9700
C7—Fe1	2.046 (3)	C17—C18	1.361 (5)
C7—H7	0.9300	C17—N1	1.365 (4)
C8—C9	1.402 (4)	C17—H17	0.9300
C8—Fe1	2.045 (3)	C18—N2	1.379 (4)
C8—H8	0.9300	C18—H18	0.9300
C9—C10	1.416 (4)	C19—N2	1.312 (4)
C9—Fe1	2.038 (3)	C19—N1	1.351 (4)
C9—H9	0.9300	C19—H19	0.9300
			
C2—C1—C5	108.2 (3)	H13A—C13—H13B	107.7
C2—C1—Fe1	70.42 (18)	C13—C14—C15	115.1 (3)
C5—C1—Fe1	70.09 (17)	C13—C14—H14A	108.5
C2—C1—H1	125.9	C15—C14—H14A	108.5
C5—C1—H1	125.9	C13—C14—H14B	108.5
Fe1—C1—H1	125.2	C15—C14—H14B	108.5
C1—C2—C3	107.6 (3)	H14A—C14—H14B	107.5
C1—C2—Fe1	69.08 (18)	C14—C15—C16	114.0 (3)
C3—C2—Fe1	69.98 (17)	C14—C15—H15A	108.8
C1—C2—H2	126.2	C16—C15—H15A	108.8
C3—C2—H2	126.2	C14—C15—H15B	108.8
Fe1—C2—H2	126.3	C16—C15—H15B	108.8
C4—C3—C2	107.9 (3)	H15A—C15—H15B	107.7
C4—C3—Fe1	69.48 (17)	N1—C16—C15	114.0 (3)
C2—C3—Fe1	69.61 (17)	N1—C16—H16A	108.8
C4—C3—H3	126.0	C15—C16—H16A	108.8
C2—C3—H3	126.0	N1—C16—H16B	108.8
Fe1—C3—H3	126.4	C15—C16—H16B	108.8
C5—C4—C3	108.5 (3)	H16A—C16—H16B	107.6
C5—C4—Fe1	69.89 (18)	C18—C17—N1	106.6 (3)
C3—C4—Fe1	70.25 (18)	C18—C17—H17	126.7
C5—C4—H4	125.8	N1—C17—H17	126.7
C3—C4—H4	125.8	C17—C18—N2	109.8 (3)
Fe1—C4—H4	125.7	C17—C18—H18	125.1
C4—C5—C1	107.8 (3)	N2—C18—H18	125.1
C4—C5—Fe1	69.94 (18)	N2—C19—N1	112.9 (3)
C1—C5—Fe1	69.19 (17)	N2—C19—H19	123.6
C4—C5—H5	126.1	N1—C19—H19	123.6
C1—C5—H5	126.1	C19—N1—C17	106.1 (3)
Fe1—C5—H5	126.3	C19—N1—C16	126.3 (3)
C7—C6—C10	108.3 (3)	C17—N1—C16	127.5 (3)
C7—C6—Fe1	69.80 (18)	C19—N2—C18	104.6 (3)
C10—C6—Fe1	70.11 (16)	C1—Fe1—C4	68.03 (13)
C7—C6—H6	125.8	C1—Fe1—C9	121.05 (14)
C10—C6—H6	125.8	C4—Fe1—C9	122.84 (13)
Fe1—C6—H6	125.8	C1—Fe1—C5	40.72 (13)
C8—C7—C6	107.7 (3)	C4—Fe1—C5	40.18 (12)
C8—C7—Fe1	69.76 (19)	C9—Fe1—C5	106.32 (14)
C6—C7—Fe1	69.52 (17)	C1—Fe1—C6	124.39 (14)
C8—C7—H7	126.2	C4—Fe1—C6	159.09 (13)
C6—C7—H7	126.2	C9—Fe1—C6	67.90 (12)
Fe1—C7—H7	126.1	C5—Fe1—C6	159.95 (13)
C9—C8—C7	108.1 (3)	C1—Fe1—C2	40.50 (13)
C9—C8—Fe1	69.68 (17)	C4—Fe1—C2	67.94 (13)
C7—C8—Fe1	69.85 (18)	C9—Fe1—C2	157.40 (14)
C9—C8—H8	126.0	C5—Fe1—C2	68.11 (14)
C7—C8—H8	126.0	C6—Fe1—C2	109.39 (13)
Fe1—C8—H8	126.1	C1—Fe1—C8	156.16 (15)
C8—C9—C10	109.4 (3)	C4—Fe1—C8	107.53 (13)
C8—C9—Fe1	70.17 (18)	C9—Fe1—C8	40.15 (13)
C10—C9—Fe1	70.40 (16)	C5—Fe1—C8	120.74 (14)
C8—C9—H9	125.3	C6—Fe1—C8	68.08 (13)
C10—C9—H9	125.3	C2—Fe1—C8	161.64 (14)
Fe1—C9—H9	125.7	C1—Fe1—C7	161.63 (14)
C9—C10—C6	106.6 (3)	C4—Fe1—C7	122.76 (13)
C9—C10—C11	126.4 (3)	C9—Fe1—C7	67.78 (14)
C6—C10—C11	127.1 (3)	C5—Fe1—C7	156.86 (14)
C9—C10—Fe1	69.12 (16)	C6—Fe1—C7	40.68 (12)
C6—C10—Fe1	69.14 (16)	C2—Fe1—C7	125.83 (14)
C11—C10—Fe1	127.8 (2)	C8—Fe1—C7	40.39 (13)
C12—C11—C10	115.7 (2)	C1—Fe1—C3	67.98 (13)
C12—C11—H11A	108.4	C4—Fe1—C3	40.27 (12)
C10—C11—H11A	108.4	C9—Fe1—C3	159.74 (14)
C12—C11—H11B	108.4	C5—Fe1—C3	67.75 (13)
C10—C11—H11B	108.4	C6—Fe1—C3	124.18 (13)
H11A—C11—H11B	107.4	C2—Fe1—C3	40.42 (13)
C11—C12—C13	113.9 (2)	C8—Fe1—C3	124.75 (14)
C11—C12—H12A	108.8	C7—Fe1—C3	109.58 (13)
C13—C12—H12A	108.8	C1—Fe1—C10	106.89 (13)
C11—C12—H12B	108.8	C4—Fe1—C10	158.58 (13)
C13—C12—H12B	108.8	C9—Fe1—C10	40.48 (11)
H12A—C12—H12B	107.7	C5—Fe1—C10	122.47 (13)
C14—C13—C12	113.3 (3)	C6—Fe1—C10	40.75 (11)
C14—C13—H13A	108.9	C2—Fe1—C10	122.62 (13)
C12—C13—H13A	108.9	C8—Fe1—C10	68.22 (13)
C14—C13—H13B	108.9	C7—Fe1—C10	68.52 (12)
C12—C13—H13B	108.9	C3—Fe1—C10	159.11 (13)
			
C5—C1—C2—C3	0.6 (4)	C1—C5—Fe1—C7	169.6 (3)
Fe1—C1—C2—C3	−59.6 (2)	C4—C5—Fe1—C3	−37.51 (19)
C5—C1—C2—Fe1	60.2 (2)	C1—C5—Fe1—C3	81.6 (2)
C1—C2—C3—C4	−0.1 (4)	C4—C5—Fe1—C10	163.07 (18)
Fe1—C2—C3—C4	−59.1 (2)	C1—C5—Fe1—C10	−77.8 (2)
C1—C2—C3—Fe1	59.0 (2)	C7—C6—Fe1—C1	165.47 (19)
C2—C3—C4—C5	−0.5 (3)	C10—C6—Fe1—C1	−75.2 (2)
Fe1—C3—C4—C5	−59.7 (2)	C7—C6—Fe1—C4	44.1 (4)
C2—C3—C4—Fe1	59.2 (2)	C10—C6—Fe1—C4	163.4 (3)
C3—C4—C5—C1	0.8 (3)	C7—C6—Fe1—C9	−81.2 (2)
Fe1—C4—C5—C1	−59.0 (2)	C10—C6—Fe1—C9	38.15 (17)
C3—C4—C5—Fe1	59.9 (2)	C7—C6—Fe1—C5	−158.0 (4)
C2—C1—C5—C4	−0.9 (3)	C10—C6—Fe1—C5	−38.7 (5)
Fe1—C1—C5—C4	59.5 (2)	C7—C6—Fe1—C2	122.9 (2)
C2—C1—C5—Fe1	−60.4 (2)	C10—C6—Fe1—C2	−117.8 (2)
C10—C6—C7—C8	−0.2 (3)	C7—C6—Fe1—C8	−37.70 (19)
Fe1—C6—C7—C8	59.5 (2)	C10—C6—Fe1—C8	81.6 (2)
C10—C6—C7—Fe1	−59.7 (2)	C10—C6—Fe1—C7	119.3 (3)
C6—C7—C8—C9	0.0 (4)	C7—C6—Fe1—C3	80.3 (2)
Fe1—C7—C8—C9	59.4 (2)	C10—C6—Fe1—C3	−160.38 (19)
C6—C7—C8—Fe1	−59.4 (2)	C7—C6—Fe1—C10	−119.3 (3)
C7—C8—C9—C10	0.2 (3)	C3—C2—Fe1—C1	119.0 (3)
Fe1—C8—C9—C10	59.7 (2)	C1—C2—Fe1—C4	−81.5 (2)
C7—C8—C9—Fe1	−59.5 (2)	C3—C2—Fe1—C4	37.5 (2)
C8—C9—C10—C6	−0.3 (3)	C1—C2—Fe1—C9	41.7 (4)
Fe1—C9—C10—C6	59.3 (2)	C3—C2—Fe1—C9	160.7 (3)
C8—C9—C10—C11	178.2 (3)	C1—C2—Fe1—C5	−38.1 (2)
Fe1—C9—C10—C11	−122.2 (3)	C3—C2—Fe1—C5	80.9 (2)
C8—C9—C10—Fe1	−59.6 (2)	C1—C2—Fe1—C6	120.7 (2)
C7—C6—C10—C9	0.3 (3)	C3—C2—Fe1—C6	−120.3 (2)
Fe1—C6—C10—C9	−59.26 (19)	C1—C2—Fe1—C8	−160.6 (4)
C7—C6—C10—C11	−178.2 (3)	C3—C2—Fe1—C8	−41.6 (5)
Fe1—C6—C10—C11	122.2 (3)	C1—C2—Fe1—C7	163.14 (19)
C7—C6—C10—Fe1	59.6 (2)	C3—C2—Fe1—C7	−77.9 (2)
C9—C10—C11—C12	164.6 (3)	C1—C2—Fe1—C3	−119.0 (3)
C6—C10—C11—C12	−17.2 (4)	C1—C2—Fe1—C10	77.4 (2)
Fe1—C10—C11—C12	74.0 (3)	C3—C2—Fe1—C10	−163.61 (19)
C10—C11—C12—C13	−178.3 (3)	C9—C8—Fe1—C1	45.3 (4)
C11—C12—C13—C14	−174.7 (3)	C7—C8—Fe1—C1	164.5 (3)
C12—C13—C14—C15	−177.4 (3)	C9—C8—Fe1—C4	120.5 (2)
C13—C14—C15—C16	178.1 (3)	C7—C8—Fe1—C4	−120.3 (2)
C14—C15—C16—N1	68.2 (3)	C7—C8—Fe1—C9	119.2 (3)
N1—C17—C18—N2	−0.7 (4)	C9—C8—Fe1—C5	78.6 (2)
N2—C19—N1—C17	0.9 (3)	C7—C8—Fe1—C5	−162.19 (19)
N2—C19—N1—C16	−177.2 (3)	C9—C8—Fe1—C6	−81.3 (2)
C18—C17—N1—C19	−0.1 (3)	C7—C8—Fe1—C6	37.96 (18)
C18—C17—N1—C16	177.9 (3)	C9—C8—Fe1—C2	−166.9 (4)
C15—C16—N1—C19	91.5 (4)	C7—C8—Fe1—C2	−47.7 (5)
C15—C16—N1—C17	−86.2 (4)	C9—C8—Fe1—C7	−119.2 (3)
N1—C19—N2—C18	−1.3 (4)	C9—C8—Fe1—C3	161.50 (19)
C17—C18—N2—C19	1.2 (4)	C7—C8—Fe1—C3	−79.3 (2)
C2—C1—Fe1—C4	81.3 (2)	C9—C8—Fe1—C10	−37.19 (18)
C5—C1—Fe1—C4	−37.42 (19)	C7—C8—Fe1—C10	82.0 (2)
C2—C1—Fe1—C9	−162.62 (19)	C8—C7—Fe1—C1	−160.0 (4)
C5—C1—Fe1—C9	78.7 (2)	C6—C7—Fe1—C1	−41.1 (5)
C2—C1—Fe1—C5	118.7 (3)	C8—C7—Fe1—C4	78.3 (2)
C2—C1—Fe1—C6	−79.5 (2)	C6—C7—Fe1—C4	−162.83 (17)
C5—C1—Fe1—C6	161.76 (18)	C8—C7—Fe1—C9	−37.44 (18)
C5—C1—Fe1—C2	−118.7 (3)	C6—C7—Fe1—C9	81.47 (19)
C2—C1—Fe1—C8	165.0 (3)	C8—C7—Fe1—C5	42.0 (4)
C5—C1—Fe1—C8	46.3 (4)	C6—C7—Fe1—C5	160.9 (3)
C2—C1—Fe1—C7	−48.2 (5)	C8—C7—Fe1—C6	−118.9 (3)
C5—C1—Fe1—C7	−167.0 (4)	C8—C7—Fe1—C2	163.3 (2)
C2—C1—Fe1—C3	37.7 (2)	C6—C7—Fe1—C2	−77.8 (2)
C5—C1—Fe1—C3	−81.0 (2)	C6—C7—Fe1—C8	118.9 (3)
C2—C1—Fe1—C10	−120.8 (2)	C8—C7—Fe1—C3	121.0 (2)
C5—C1—Fe1—C10	120.5 (2)	C6—C7—Fe1—C3	−120.06 (19)
C5—C4—Fe1—C1	37.91 (19)	C8—C7—Fe1—C10	−81.2 (2)
C3—C4—Fe1—C1	−81.4 (2)	C6—C7—Fe1—C10	37.71 (17)
C5—C4—Fe1—C9	−75.8 (2)	C4—C3—Fe1—C1	81.5 (2)
C3—C4—Fe1—C9	164.9 (2)	C2—C3—Fe1—C1	−37.8 (2)
C3—C4—Fe1—C5	−119.3 (3)	C2—C3—Fe1—C4	−119.3 (3)
C5—C4—Fe1—C6	168.5 (3)	C4—C3—Fe1—C9	−39.2 (5)
C3—C4—Fe1—C6	49.2 (4)	C2—C3—Fe1—C9	−158.5 (4)
C5—C4—Fe1—C2	81.8 (2)	C4—C3—Fe1—C5	37.42 (19)
C3—C4—Fe1—C2	−37.6 (2)	C2—C3—Fe1—C5	−81.9 (2)
C5—C4—Fe1—C8	−117.2 (2)	C4—C3—Fe1—C6	−160.95 (19)
C3—C4—Fe1—C8	123.5 (2)	C2—C3—Fe1—C6	79.7 (2)
C5—C4—Fe1—C7	−158.9 (2)	C4—C3—Fe1—C2	119.3 (3)
C3—C4—Fe1—C7	81.8 (2)	C4—C3—Fe1—C8	−75.4 (2)
C5—C4—Fe1—C3	119.3 (3)	C2—C3—Fe1—C8	165.2 (2)
C5—C4—Fe1—C10	−42.3 (4)	C4—C3—Fe1—C7	−117.9 (2)
C3—C4—Fe1—C10	−161.6 (3)	C2—C3—Fe1—C7	122.7 (2)
C8—C9—Fe1—C1	−160.4 (2)	C4—C3—Fe1—C10	161.1 (3)
C10—C9—Fe1—C1	79.4 (2)	C2—C3—Fe1—C10	41.8 (4)
C8—C9—Fe1—C4	−77.9 (2)	C9—C10—Fe1—C1	−118.3 (2)
C10—C9—Fe1—C4	161.90 (18)	C6—C10—Fe1—C1	123.5 (2)
C8—C9—Fe1—C5	−118.6 (2)	C11—C10—Fe1—C1	2.1 (3)
C10—C9—Fe1—C5	121.24 (19)	C9—C10—Fe1—C4	−45.6 (4)
C8—C9—Fe1—C6	81.8 (2)	C6—C10—Fe1—C4	−163.8 (3)
C10—C9—Fe1—C6	−38.40 (17)	C11—C10—Fe1—C4	74.9 (4)
C8—C9—Fe1—C2	169.3 (3)	C6—C10—Fe1—C9	−118.2 (3)
C10—C9—Fe1—C2	49.1 (4)	C11—C10—Fe1—C9	120.5 (3)
C10—C9—Fe1—C8	−120.2 (3)	C9—C10—Fe1—C5	−76.6 (2)
C8—C9—Fe1—C7	37.66 (19)	C6—C10—Fe1—C5	165.29 (19)
C10—C9—Fe1—C7	−82.49 (19)	C11—C10—Fe1—C5	43.9 (3)
C8—C9—Fe1—C3	−48.8 (5)	C9—C10—Fe1—C6	118.2 (3)
C10—C9—Fe1—C3	−169.0 (3)	C11—C10—Fe1—C6	−121.4 (3)
C8—C9—Fe1—C10	120.2 (3)	C9—C10—Fe1—C2	−159.8 (2)
C4—C5—Fe1—C1	−119.1 (3)	C6—C10—Fe1—C2	82.0 (2)
C1—C5—Fe1—C4	119.1 (3)	C11—C10—Fe1—C2	−39.3 (3)
C4—C5—Fe1—C9	121.94 (19)	C9—C10—Fe1—C8	36.90 (19)
C1—C5—Fe1—C9	−118.9 (2)	C6—C10—Fe1—C8	−81.3 (2)
C4—C5—Fe1—C6	−168.0 (3)	C11—C10—Fe1—C8	157.4 (3)
C1—C5—Fe1—C6	−48.9 (5)	C9—C10—Fe1—C7	80.5 (2)
C4—C5—Fe1—C2	−81.3 (2)	C6—C10—Fe1—C7	−37.65 (18)
C1—C5—Fe1—C2	37.86 (19)	C11—C10—Fe1—C7	−159.0 (3)
C4—C5—Fe1—C8	80.7 (2)	C9—C10—Fe1—C3	169.3 (3)
C1—C5—Fe1—C8	−160.12 (19)	C6—C10—Fe1—C3	51.2 (4)
C4—C5—Fe1—C7	50.4 (4)	C11—C10—Fe1—C3	−70.2 (5)

### Hydrogen-bond geometry (Å, °)

**Table:**

*D*—H···*A*	*D*—H	H···*A*	*D*···*A*	*D*—H···*A*
C19—H19···N2^i^	0.93	2.58	3.399 (5)	147

Symmetry codes: (i) −*x*+1/2, *y*+1/2, *z*.
